# Inter-α-inhibitor Impairs TSG-6-induced Hyaluronan Cross-linking*

**DOI:** 10.1074/jbc.M113.477422

**Published:** 2013-09-04

**Authors:** Natalia S. Baranova, Simon J. Foulcer, David C. Briggs, Viranga Tilakaratna, Jan J. Enghild, Caroline M. Milner, Anthony J. Day, Ralf P. Richter

**Affiliations:** From the ‡ Biosurfaces Unit, CIC biomaGUNE, 20009 Donostia-San Sebastian, Spain,; the § Wellcome Trust Centre for Cell Matrix Research and; the ‖ Faculty of Life Sciences, University of Manchester, Manchester M13 9PT, United Kingdom,; the ¶ Department of Molecular Biology and Genetics, University of Aarhus, 8000 Aarhus C, Denmark,; the ** Max Planck Institute for Intelligent Systems, 70569 Stuttgart, Germany, and; the ‡‡ Department of Molecular Chemistry, Joseph Fourier University, 38041 Grenoble Cedex 9, France

**Keywords:** Carbohydrate-binding Protein, Extracellular Matrix, Extracellular Matrix Proteins, Glycosaminoglycan, Multifunctional Protein, Protein Complexes, Protein Self-assembly, Protein-Protein Interactions, Hyaluronan, Supramolecular Interactions

## Abstract

Under inflammatory conditions and in the matrix of the cumulus-oocyte complex, the polysaccharide hyaluronan (HA) becomes decorated covalently with heavy chains (HCs) of the serum glycoprotein inter-α-inhibitor (IαI). This alters the functional properties of the HA as well as its structural role within extracellular matrices. The covalent transfer of HCs from IαI to HA is catalyzed by TSG-6 (tumor necrosis factor-stimulated gene-6), but TSG-6 is also known as a HA cross-linker that induces condensation of the HA matrix. Here, we investigate the interplay of these two distinct functions of TSG-6 by studying the ternary interactions of IαI and TSG-6 with well defined films of end-grafted HA chains. We demonstrate that TSG-6-mediated cross-linking of HA films is impaired in the presence of IαI and that this effect suppresses the TSG-6-mediated enhancement of HA binding to CD44-positive cells. Furthermore, we find that the interaction of TSG-6 and IαI in the presence of HA gives rise to two types of complexes that independently promote the covalent transfer of heavy chains to HA. One type of complex interacts very weakly with HA and is likely to correspond to the previously reported covalent HC·TSG-6 complexes. The other type of complex is novel and binds stably but noncovalently to HA. Prolonged incubation with TSG-6 and IαI leads to HA films that contain, in addition to covalently HA-bound HCs, several tightly but noncovalently bound molecular species. These findings have important implications for understanding how the biological activities of TSG-6 are regulated, such that the presence or absence of IαI will dictate its function.

## Teaser

**Background:** Inflammation/ovulation-associated protein TSG-6 performs multiple functions in hyaluronan (HA)-rich extracellular matrices.

**Results:** Inter-α-inhibitor (IαI) affects HA-TSG-6 interactions and enhancement of cell adhesion while promoting covalent complex formation between IαI heavy chains and HA.

**Conclusion:** IαI dictates TSG-6 activity and remodels HA matrix properties.

**Significance:** These results provide novel insights into the regulation of HA-protein interactions and assembly of biologically important extracellular matrices.

## Introduction

Hyaluronan (HA)^4^ is the main non-protein component of the extracellular matrix of vertebrates, and it plays an important role in many physiological and pathological processes, such as inflammation and ovulation (1, 2). HA is a high molecular weight glycosaminoglycan composed of repeating disaccharides of *N*-acetylglucosamine and glucuronic acid. The linear polysaccharide is flexible and can adopt various conformations.

Proteins that bind to hyaluronan can drive conformational changes and thereby remodel the morphology and physicochemical properties of HA-rich extracellular matrices (3–5). For example, HA can be modified with heavy chains (HCs) of inter-α-inhibitor (IαI). The ensuing covalent HA·HC complex is also known as serum-derived hyaluronan-associated protein (SHAP) complex (6, 7).

This modification is the only naturally occurring covalent modification of HA known to date. It was shown to be critical for the expansion of the cumulus-oocyte complex (COC) matrix around oocytes (8, 9). Correct assembly of the HA matrix around oocytes is crucial for successful ovulation and fertilization (8–13). HA·HC complexes were also found under inflammatory conditions in synovial fluid of arthritis patients (7, 14). HA·HC extracts from rheumatoid synovial fluid can undergo gelation at pH 4.5 (15) and can form macromolecular aggregates that are more adhesive for leukocytes via their enhanced interaction with CD44 (7, 16). Airway smooth muscle cells in response to poly(I:C) were also demonstrated to produce HA·HC cable like complexes with enhanced leukocyte binding (17). It has been hypothesized that HCs mediate cross-linking of HA and thereby induce the observed changes in the morphology, rheological, and cell binding properties of HA assemblies (5, 7, 18).

HCs are subunits of IαI, a serum proteoglycan that consists of heavy chain 1 (HC1), heavy chain 2 (HC2), and bikunin, a serine protease inhibitor, held together by a chondroitin 4-sulfate (C4S) chain (19). Each HC is linked to C4S via an ester bond between the C-terminal Asp residue of the HC and the C-6 of *N*-acetylgalactosamine in C4S, which is itself attached to the bikunin core protein through a standard glycosaminoglycan linkage (20).

The transfer of HCs from IαI onto HA is mediated by TSG-6 (secreted product of tumor necrosis factor-stimulated gene-6) in two sequential transesterification reactions (21). First, a covalent complex is formed between TSG-6 and either HC1 or HC2 (21–23) followed by the transfer of the HC from this HC·TSG-6 intermediate onto the C-6 hydroxyl of *N*-acetylglucosamine of HA (6). Here TSG-6 acts as a catalyst because its release from the HC1·TSG-6 or HC2·TSG-6 complexes allows it to be recycled for a new reaction with IαI (21).

There is evidence that intact IαI (or IαΙ-related species that contain at least one ester bond (24)) is required for this reaction (8, 25). Bikunin null mice, which have impaired female fertility (8, 26) while expressing HCs, did not assemble an IαI molecule and failed to form HA·HC complexes (8); ovulation/fertilization was rescued by intraperitoneal administration of purified IαI, but not bikunin alone (8). Furthermore, partial proteolysis of bikunin did not influence the transfer of HCs onto TSG-6 (25), indicating that the function of bikunin is to provide the C4S chain where ester bonds with HCs can be formed; the energy stored in the ester bonds (during biosynthesis) is used to drive subsequent transfer of HCs onto TSG-6 and ultimately their attachment to HA (21). Both of these transesterification steps require the presence of divalent cations (21, 25), for which Ca^2+^ and Mg^2+^/Mn^2+^ ions have been implicated as being involved (21, 27).

In addition to its enzymatic function, TSG-6 is well established as an HA-binding protein (28, 29). It is composed mainly of two contiguous domains (30): a Link module, where the HA-binding groove is located (31–33), and a CUB module (Protein Data Bank code 2WNO) (34). Recently, we have shown that full-length TSG-6 alone can cross-link HA and that TSG-6 oligomers that are induced by the binding of TSG-6 to HA constitute the cross-linking entities (4). We also showed that the TSG-6-mediated cross-linking can induce a drastic condensation of an HA network.

Considering that both TSG-6 and HA can participate in two distinct processes—HC transfer on the one hand and TSG-6-mediated HA cross-linking on the other—raises the question of how these two processes influence each other. This is perhaps of particular relevance for COC matrix expansion. Here the expression of TSG-6 mRNA is detectable within 2 h after the induction of ovulation, in cumulus and granulosa cells in ovarian follicles harboring an oocyte (35, 36), which is a similar time scale to the initiation of HA biosynthesis (37). IαI is thought to diffuse rapidly into the follicle after initiation of ovulation; COC matrix-associated IαI was reported to increase over time, and HA·HC complexes are typically detected after 6 h (38–41). *TSG-6*^−/−^ mice (like bikunin null animals (8, 26)) were unable to assemble a functional COC matrix and had a phenotype that correlates with the total absence of HA·HC complexes (9); however, administration of recombinant TSG-6 in the presence of IαI-containing serum was able to rescue COC matrix expansion.

To directly study how the presence of IαI affects the binding of TSG-6 to HA and to understand how the HA-cross-linking and enzymatic activities of TSG-6 influence each other, we designed *in vitro* binding assays in which TSG-6 and/or IαI can interact with well defined films of HA in controlled sequence and concentrations. We demonstrate that the HA binding properties of TSG-6 are impaired in the presence of IαI, and as a consequence the TSG-6-mediated condensation of HA does not occur. We also provide novel insight into the kinetics of the TSG-6-mediated enzymatic transfer of HCs from IαI to HA and show that HA·HC complex formation is accompanied by the incorporation of tightly but noncovalently bound protein material into the HA matrix.

## EXPERIMENTAL PROCEDURES

### 

#### 

##### Protein and Hyaluronan Preparations

IαI was purified from human serum as described previously (19). Full-length recombinant human TSG-6 (rhTSG-6; 30.1 kDa) was expressed in *Drosophila* Schneider 2 cells and purified as described previously (42).

Recombinant human heavy chains 1, 2, and 3 (rHC1, rHC2, and rHC3) were expressed in *Escherichia coli* “SHuffle” cells (New England Biolabs). Codon optimized genes encoding the mature protein sequences (rHC1, Uniprot P19827 amino acid residues 35–672; rHC2, Uniprot P19823 amino acid residues 55–702; and rHC3, Uniprot Q06033 amino acid residues 35–651) were cloned into pET-45b+, using BamHI and HindIII restriction sites, by Genscript USA, Inc. Transformed cells were cultured in Terrific Broth at 30 °C, and protein expression was induced by addition of isopropyl β-d-thiogalactopyranoside to 0.5 mm at an *A*_600 nm_ of 0.6. Cells were harvested at 16 h postinduction, at either 30 °C (rHC1 and rHC3) or 20 °C (rHC2), and lysed by sonication. Protein purification was achieved by Ni^2+^ ion affinity chromatography, followed by HiTrap heparin affinity chromatography (rHC1 and rHC2) or anion exchange (rHC3) and size exclusion chromatography on a Superdex-200 column. Purity was assayed by SDS-PAGE (see “Results”) and electrospray ionization mass spectrometry. The latter analysis gave molecular masses of 73,802.8 Da (rHC1), 74,842.4 Da (rHC2), and 71,754.4 Da (rHC3), which are in good agreement (within 3 Da) with the theoretical masses expected for these constructs in a nonreduced (*i.e.*, disulfide bonded) form and missing the N-terminal methionine residue (*i.e.*, 73,805.4, 74,842.7, and 71,752.5 Da, respectively). rHC1 has been crystallized, and its structure has been determined to 2.5 Å,^5^ indicating that the method of HC expression used here leads to folded protein.

Lyophilized HA, biotinylated at its reducing end and with well defined molecular masses of 1,083 ± 53 or 837 ± 54 kDa (*i.e.*, two different batches of Select-HA B1000) was purchased from Hyalose (Oklahoma City, OK). For reconstitution, HA was taken up in ultrapure water at a stock concentration of 1 mg/ml, gently shaken overnight, aliquoted, and stored at −20 °C.

A HEPES buffer (150 mm NaCl, 10 mm HEPES at pH 7.4, 3 mm NaN_3_, 2 mm CaCl_2_, 5 mm MgCl_2_ in ultrapure water) was used throughout all measurements. Protein and HA solutions at their final concentrations were prepared in this buffer.

##### Assembly of Films of End-grafted HA on a Biotin-functionalized Surface Coating

**FIGURE 1.**
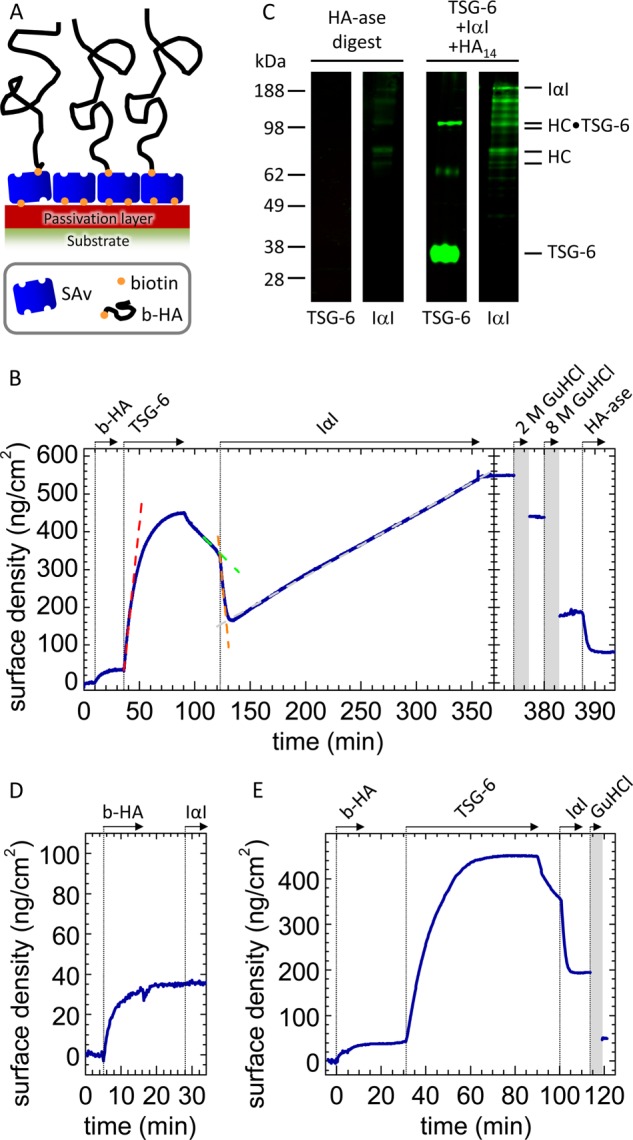
**IαI partially impairs TSG-6 binding to HA.** *A*, architecture of end-grafted hyaluronan films. A biotin-functionalized passivation layer was immobilized on a gold support, followed by the formation of a dense SAv layer. HA chains were grafted via a biotin functionality at their reducing end to SAv. The thickness of the passivation layer and the size of SAv are drawn to scale; the thickness of the HA brush and the mean distances between HA anchors are reduced by 10–20-fold for illustrative purposes. *B*, sequential incubation assay by ellipsometry. The start and duration of each incubation step with different samples are indicated with *solid arrows*; after each incubation step, the solution phase was replaced by buffer. First, a biotinylated HA (*b-HA*, 837 kDa) film was formed and incubated with 0.3 μm TSG-6. After binding equilibrium had been established, excess TSG-6 was removed from the bulk solution. Addition of IαI at 1 μm strongly enhanced TSG-6 displacement from the HA film (*dashed lines* are linear fits to the data at selected times). A fraction of 38% of the TSG-6 mass was not displaced by IαI, and prolonged incubation led to renewed incorporation of protein material at a constant rate. Proteins were eluted by sequential incubation with 2 and 8 m GuHCl (*shaded* in *gray*; elution process not monitored by ellipsometry), and the remainder of the film was digested with HA-ase. The residual response after HA-ase treatment, which we suggest may be due to limited accessibility of HA for digestion, was not always observed. *C*, Western blots of the HA-ase digest. No TSG-6 was detected. The strongest bands for IαI, at ∼80 kDa, are assigned to HC1 and HC2. Based on the ellipsometry data, the amount of total digested protein material used per lane was estimated to be ∼10 ng. The control reaction mix of TSG-6, HA_14_, and IαI is expected to contain a total amount of 100 ng of TSG-6 and 25 ng of IαI, and the detection limits are estimated to be ∼5 ng for TSG-6 and 0.5 ng for IαI. Western blots of the fractions eluted with 2 and 8 m GuHCl were essentially identical to the blots shown in Fig. 3*B* and are omitted here for the sake of clarity/brevity. *D*, control showing that 1 μm IαI does not bind to HA films. *E*, the protein fraction retained after brief exposure of a TSG-6-loaded HA films to IαI is noncovalently bound. A sequential incubation assay was performed similar to *B*, but the incubation with IαI was kept short (10 min). After incubation with 6 m GuHCl, the surface density returned to the same level as for a pure HA film, indicating that essentially all protein was stably but noncovalently bound.

HA films were prepared as described previously (4) (see Fig. 1*A*). Briefly, a dense streptavidin (SAv) monolayer was formed by exposure of 50 μg/ml SAv (30 min) to a gold surface that had been functionalized with a biotinylated oligoethylene glycol monolayer. Biotinylated HA was then grafted to the SAv monolayer by incubation of 10 μg/ml HA solution. The assembly steps were performed at room temperature, typically 23 °C.

The HA grafting density was set to 30 ± 10 ng/cm^2^ by adjusting the incubation time in ellipsometry measurements. This corresponds to a mean anchor distance of 81 ± 14 nm between neighboring HA grafting points. End-grafted HA at this grafting density forms a so-called brush of entangled HA chains that are weakly stretched in the direction normal to the surface (43, 44). For polymer brushes, the mesh size in the film is predicted to be as large as the mean anchor distance (45). Therefore, molecules such as TSG-6 and IαI should be able to diffuse rapidly in and out of the HA film.

In the measurements by colloidal probe reflection interference contrast microscopy (RICM), the incubation time was kept at 2 h. From the measured film thickness, we estimate the areal surface density to be 50 ± 15 ng/cm^2^ (44). To release noncovalently bound material, the HA film was treated with 2 or 8 m guanidine hydrochloride (GuHCl) for 5 min. The HA films are stable to this treatment (4). To this end, GuHCl was dissolved at 8 m in ultrapure water, with the pH adjusted to 7.4 (with HCl). To obtain lower concentrations, the GuHCl solution was diluted in HEPES buffer.

##### Co-incubation Assay

IαI and TSG-6 were premixed at concentrations of 9.8 and 3.3 μm, respectively, at 23 °C for desired times (1 to 120 min). The premixed solution was then injected into the measurement cell that contained buffer and the HA film (at room temperature, typically 23 °C). Rapid mixing generated final concentrations in the soluble phase of 1 μm IαI and 0.3 μm TSG-6. Alternatively, IαI and TSG-6 were sequentially injected into the measurement cell at the same final concentrations. In this case, we refer to 0 min of premixing time. In all co-incubation assays, the HA grafting density was fixed to 33 ± 10 ng/cm^2^.

##### In Situ Ellipsometry

Ellipsometry measures changes in the polarization of light upon reflection at a planar surface. We employed ellipsometry *in situ* on gold-coated silicon wafers as substrates that were installed in a custom-built open cuvette with continuously stirred sample solution (∼150 μl), to quantify adsorbed biomolecular masses in a time-resolved manner (46).

##### Surface Plasmon Resonance

Surface plasmon resonance data were acquired using a BiaCore 3000 (GE Healthcare). rHC1, rHC2, or rHC3 was immobilized at a concentration of 10 μg/ml in either 10 mm sodium acetate, pH 5.5 (rHC1 and rHC2) or pH 4.0 (rHC3), to a C1 chip (GE Healthcare) using Sulfo-NHS/EDC amine coupling. Immobilization contact times were adjusted to give ∼1,000 response units for all three proteins. Experiments were conducted in HEPES-buffered saline with 0.5% (v/v) Tween 20 (HBS-T) either with 10 mm EDTA added or 1 mm each of CaCl_2_ and MgCl_2_. All experiments were conducted with a flow rate of 50 μl/min. TSG-6 was used as analyte at concentrations ranging from 6.25 to 200 nm. Data were fitted to a 1:1 Langmuir model using BiaEval software.

##### Colloidal Probe Reflection Interference Contrast Microscopy (RICM)

This microinterferometic technique measures the height at which a colloidal probe hovers above a transparent planar substrate with a resolution of a few nanometers over a range of ∼1 μm. RICM was used to measure the thickness of HA films that were assembled on gold-coated glass cover slips, using a custom-built measurement cell (4, 47).

##### Western Blotting

Samples of ∼150 μl volume were extracted from the ellipsometry cuvette and stored frozen in aliquots of 50 μl until further use. One aliquot per lane was used for Western blot analysis; protein was recovered using 10 μl of StrataClean resin (Agilent Technologies) according to the manufacturer's instructions, and following a wash with water, the resin was analyzed by SDS-PAGE on 4–12% (w/v) NuPAGE Bis-Tris polyacrylamide gels (Invitrogen) after boiling in SDS loading buffer containing β-mercaptoethanol. A rabbit anti-human polyclonal antibody against IαI (DAKO), 1:20,000 dilution, was used to screen for IαI and its subunits, and the anti-TSG-6 antibody RAH-1 (48) at 1:1,000 was used to detect TSG-6; bands were visualized with a goat anti-rabbit LI-COR Odyssey infrared secondary antibody using a LI-COR Odyssey imaging system. As a control 2 μg of TSG-6 was incubated with 8 μg of IαI and 1 μg of HA_14_ in 20 mm HEPES-HCl (pH 7.5), 150 mm NaCl, and 5 mm MgCl_2_ in a total volume of 25 μl for 2 h at 4 °C. For TSG-6 blots, 1.25 μl of the reaction mix was used per lane (to get ∼100 ng of TSG-6), and for IαI blots, the reaction mix was diluted 1:10 and 0.78 μl were used per lane (to get ∼25 ng of IαI).

##### Fluoresceinamine Labeling of HA

Fluorescently labeled HA was produced essentially as described by De Belder and Wik (49). In brief, 10 mg of polymeric HA (HyluMed Medical Grade; Genzyme Corporation; ∼1.5 MDa) was resuspended in 8 ml of H_2_O by incubating at 4 °C overnight and then added to 4 ml of Me_2_SO. Fluoresceinamine (5 mg) was combined with 5 μl of acetaldehyde, 5 μl of cyclohexyl isocyanide, and 300 μl of Me_2_SO; combined with the HA solution; and gently stirred at room temperature for 5 h. The reaction mixture was transferred to 160 ml of ice-cold ethanol, and the labeled HA was precipitated by adding ∼2 ml of saturated NaCl solution. The precipitate was collected by centrifugation (3,000 × *g*, 10 min) and resuspended in 10 ml of H_2_O, and the ethanol precipitation and centrifugation steps were repeated. The fluorescein-labeled HA (fl-HA) was resuspended in 20 ml of H_2_O and dialyzed overnight against 2 liters of H_2_O containing 0.05% (w/v) sodium azide.

##### Flow Cytometry of HA Binding to CD44+ Cells

All flow cytometry experiments were performed with a CyAn ADP analyzer coupled with Summit v4.3 software (Beckman Coulter, Fullertin, CA). Forward scatter gain was set to 3.5, and the FITC voltage gate was set to 530 V. The level of fluorescence was quantified as the mean fluorescence intensity of ∼10,000 cells.

AKR1 and AKR1/CD44+ cell lines were used for all experiments (50, 51). The AKR1 cell line is a CD44-negative T lymphoma (purified from the AKR/J mouse) that does not bind HA. The AKR1/CD44+ cells are a transfectant of the AKR1 cells containing cDNA encoding the CD44.1 allele, resulting in CD44 expression and constitutive HA binding properties.

The expression of CD44 was confirmed here using FITC-conjugated IM7 antibody (1:1,000; Abcam); all incubations were performed at 37 °C, and the cells (0.5 × 10^6^) were washed two or three times with PBS between incubations and prior to flow cytometric analysis. Binding of fl-HA to AKR1/CD44+ cells (and AKR1 cells) was assessed by incubating them together with increasing concentrations of fl-HA for 120 min; competition with unlabeled HA showed that there was only ∼4% internalization of fl-HA (at 1 μg/ml) by AKR1/CD44+ cells after 120 min incubation (data not shown). The enhancing effect of TSG-6 on HA binding to CD44 (reported in Ref. 52) was confirmed by co-incubating 0–10 μg/ml fl-HA with 0.25 μm TSG-6 prior to adding to the CD44+ cells. AKR1/CD44+ cells were also incubated with 1 μg/ml fl-HA in the absence or presence of 1 μm IαI and/or 0.3 μm TSG-6, where these incubations were done in various orders/combinations; *i.e.*, to test the effect of IαI on the TSG-6-mediated enhancement of HA binding to CD44.

## RESULTS

### 

#### 

##### IαI Partially Impairs TSG-6 Binding to HA

To understand how the HA binding properties of TSG-6 correlate with its ability to form a covalent complex with the HCs of IαI and transfer these onto HA, we designed a sequential incubation assay (Fig. 1). First, a film of end-grafted HA of molecular mass of either 1,083 or 837 kDa was assembled on a solid support (Fig. 1*A*), as described earlier (4). Second, the film was loaded with TSG-6, excess TSG-6 was removed by rinsing in buffer, and then IαI was exposed to the film. Changes in the areal surface density of HA and proteins during the assay were monitored by *in situ* ellipsometry.

We exposed the HA film to TSG-6 at a bulk concentration of 0.3 μm (Fig. 1*B*, at 36 min) until equilibrium was attained. The dissociation rate of TSG-6 after removal of excess TSG-6 from solution was relatively slow and in good quantitative agreement with the dissociation rate that we had previously reported for TSG-6 in a saturated film (4).

Importantly, subsequent addition of 1 μm IαI (Fig. 1*B*, at 122 min) strongly enhanced TSG-6 desorption: comparison of the release rates just before and after IαI injection revealed an 11-fold enhancement. A control measurement showed that IαI alone does not bind to HA (Fig. 1*D*). Hence, the formation of a complex between IαI and TSG-6 must be responsible for the displacement of TSG-6 from HA.

The IαI-induced displacement of TSG-6 was found to terminate within 10 min (Fig. 1*B*). Remarkably, a sizeable amount of protein, corresponding to approximately one-third of the maximally incorporated TSG-6 mass, remained bound to the HA film after exposure to IαI. In a similar experiment (data not shown), we incubated with 0.5 instead of 1 μm IαI, yet found the remaining fraction to be similar; in addition, a subsequent increase of the IαI concentration to 1 μm did not lead to any further release of material. This indicates that the protein remaining in the film is not simply the result of an equilibrium distribution of TSG-6 between surface-bound HA and IαI in the solution phase. Further controls showed that neither TSG-6 alone (4) nor TSG-6 in a mixture with IαI (data not shown) bound in appreciable amounts to the SAv-coated passivation layer on which the HA films were immobilized. The retained protein fraction, therefore, must be the result of a genuine interaction of TSG-6 with HA, and possibly even with IαI, that is distinct from the HA/TSG-6 interaction that can be impaired by IαI.

In yet another similar experiment (Fig. 1*E*), we found that the protein fraction that was retained 10 min after the start of incubation with IαI could be completely eluted with GuHCl. Because the HA film itself is resistant to GuHCl (4), we conclude that all bound protein was noncovalently bound at this stage. At present, we do not know whether this noncovalently bound material contains exclusively TSG-6 or also IαI (or its subunits).

##### Retained TSG-6 Promotes Slow Incorporation of IαI (or Its Subunits)

Remarkably, the areal surface density increased again upon prolonged incubation with IαI (Fig. 1*B*, beyond 132 min), at a rate of 1.7 ng/cm^2^/min. Compared with the initial binding of TSG-6 alone (Fig. 1*B*, at 36 min), the increase was ∼20-fold slower. Moreover, the binding rate was constant throughout the remaining incubation time of almost 4 h.

All incorporated protein material was stably bound: no desorption was observed after rinsing in buffer (Fig. 1*B*, at 355 min). In the presence of 2 m GuHCl (at 374 min), ∼20% of the total protein mass could be eluted, whereas exposure to 8 m GuHCl (at 380 min) resulted in the release of another 55%. This indicates that a large fraction of the protein material was very tightly bound, albeit not covalently. A significant fraction resisted even 8 m GuHCl, suggesting that some material ultimately becomes covalently incorporated. The remaining film could be partially digested with *Streptomyces* hyaluronidase (HA-ase; Fig. 1*B*, at 388 min), confirming that (at least the major proportion of) the GuHCl-resistant material is bound to HA.

The amount of noncovalently bound material at the end of the incubation process was larger than the total amount of bound material shortly after the start of incubation with IαI. This implies that at least two different interactions must contribute to the linear adsorption rate: one leading to covalently incorporated material and the other leading to tightly but noncovalently incorporated material.

TSG-6 is known to catalyze the transfer of HCs from the C4S moiety of IαI to HA chains via two sequential transesterification reactions (21, 23). We hypothesized that the covalently incorporated material corresponds to HA·HC complexes. To test this, we subjected the GuHCl eluates (not shown) and the HA-ase digest to Western blotting (Fig. 1*C*). Indeed, the strongest bands revealed with an anti-IαI antibody for the digest ran at ∼80 kDa, consistent with the sizes of HC1 and/or HC2 plus a short HA stub (21). In contrast, no bands could be detected upon staining of the digest with the anti-TSG-6 antibody RAH1, indicating that TSG-6 is not covalently incorporated into the HA films.

The areal surface density of HA-ase digestible material after the 230-min incubation process was 105 ng/cm^2^, or 35 ng/cm^2^ HA and 70 ng/cm^2^ HCs, if we assume that all HA was digested and consider that the protein fraction consists exclusively of HCs. This corresponds to an occupancy of one HC per 105 HA disaccharides or 21 HCs per HA chain of 837 kDa. The average transfer rate would be 0.06 fmol/cm^2^/s.

Considering the surface density of TSG-6 prior to incubation with IαI (310 ng/cm^2^) and the total functional surface area in our assay (0.5 cm^2^), one can estimate the total available amount of the enzyme (*i.e.*, TSG-6) throughout the HC transfer reaction to be 5 pmol. In comparison, the substrate IαI was incubated at 150 pmol, *i.e.*, in a large excess. Under these conditions, a constant rate for the enzymatic transfer would be expected, as long as TSG-6 maintains a constant enzymatic activity. In this context, the constant binding rate observed throughout the HA·HC complex formation process (Fig. 1*B*, from 132 to 355 min) receives particular significance. It implies that if the incorporation of covalently bound material (HA·HC complexes) indeed occurs at a constant rate, then the concomitant incorporation of noncovalently bound material (of currently unknown composition) must also occur at a constant rate. A simple explanation for such a response would be that both binding processes are interdependent, *i.e.*, a protein (or protein complex) is incorporated into the HC·HA film through its noncovalent interaction with the covalently attached HCs.

An additional sequential incubation assay, equivalent to that shown in Fig. 1*B* but with a lower initial TSG-6 concentration (data not shown), revealed a qualitatively similar response, *i.e.*, an initial rapid, partial release of TSG-6 followed by the binding of (partially noncovalently and partially covalently incorporated) protein material at a constant rate. The binding rate, however, was reduced, consistent with expectations for a binding process driven by a TSG-6-catalyzed HC transfer.

##### TSG-6 Can Bind Tightly but Noncovalently to HCs

**FIGURE 2.**
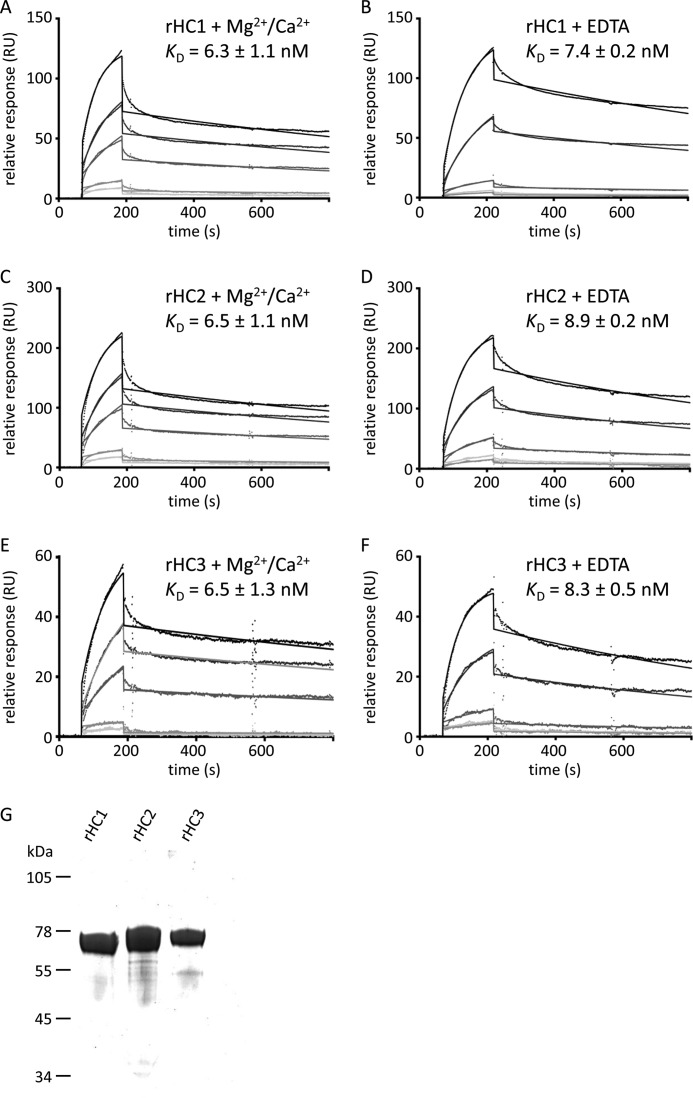
**TSG-6 interacts with high affinity with HC1, HC2, and HC3.** *A–F*, representative figures of TSG-6 (analyte) interacting with immobilized rHC1 (*A* and *B*), rHC2 (*C* and *D*), and rHC3 (*E* and *F*) shown by surface plasmon resonance in HBS-T with 1 mm MgCl_2_ and 1 mm CaCl_2_ (*A*, *C*, and *E*) or in HBS-T with 10 mm EDTA (*B*, *D*, and *F*). Raw data and curve fits (with a 1:1 Langmuir model) are shown for TSG-6 concentrations of 6.25, 12.5, 25, 50, and 100 nm. *K_D_* values were determined from the mean of three experiments ± standard deviation. Stepwise changes in response at the end of sample injection are due to RI changes upon solution exchange, modeled by BiaEval 1:1 Langmuir model fitting. Other binding models tested did not improve the fit significantly. *G*, SDS-PAGE analysis of purified recombinant rHC1, rHC2, and rHC3 (each with an N-terminal His-tag (MAHHHHHHVGTGSNDDDDKSPDP)). 3 μg of rHC1, rHC2, and rHC3, respectively, were loaded per lane and run under reducing conditions, indicating that these preparations are >95% pure.

To better understand the possible interactions between the involved proteins, we analyzed binding of recombinantly produced HC1, HC2, and HC3 (rHC1, rHC2, and rHC3), individually immobilized on a C1 chip, to TSG-6 (the analyte) by surface plasmon resonance (Fig. 2, *A–F*); SDS-PAGE of purified rHC proteins is shown in Fig. 2*G*. Analysis of the binding data revealed that all three HCs interact rapidly and strongly with TSG-6, in a divalent cation-independent manner (*i.e.*, in the presence of either Ca^2+^/Mg^2+^ or EDTA), each with a dissociation constant (*K_D_*) of 10 nm. On rates and off rates for the binding of the three HCs to TSG-6 were all very similar (*k*_on_ = 1 × 10^5^ m^−1^ s^−1^, *k*_off_ = 7 × 10^−4^ s^−1^), suggesting that they all bind (via conserved residues) to a common interaction site on TSG-6.

##### Premixing of TSG-6 and IαI Affects Protein Incorporation into HA Films

**FIGURE 3.**
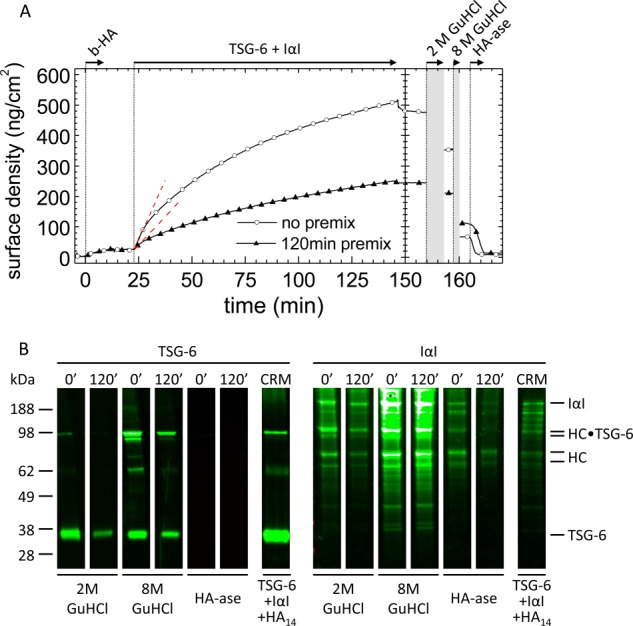
**Premixing of TSG-6 and IαI affects protein incorporation.** *A*, co-incubation assay by ellipsometry. TSG-6 and IαI were premixed for 120 min prior to exposure to the HA (837 kDa) film at final concentrations of 0.3 and 1.0 μm, respectively (*filled triangles*). Alternatively, first IαI and then TSG-6 were added to the HA film (*open circles*). The two premixing scenarios led to distinctly different binding and unbinding responses. The *dashed lines* are linear fits to the data at the start of incubation, giving initial binding rates of 1.8 and 1.1 ng/cm^2^/min, respectively. *B*, Western blots of the fractions eluted with 2 and 8 m GuHCl and of HA-ase digests. The *numbers* at the *top* of the *lanes* correspond to the premixing time in minutes; assignments of the strongest bands are indicated. Western blots for the control reaction mix (*CRM*) of TSG-6, HA_14_, and IαI are also shown. *b-HA*, biotinylated HA.

To further assess the ternary interaction between HA, TSG-6, and IαI, we performed assays in which the HA film was co-incubated with TSG-6 and IαI (Fig. 3*A*). In one assay, TSG-6 and IαI were first premixed for 120 min at room temperature, and the mixture was then added to the HA film (Fig. 3*A*, *filled triangles*). In another assay, the premixing time was minimized. To this end, we first incubated the HA film with IαI, because these two species do not interact (Fig. 1*D*) and then added TSG-6 (Fig. 3*A*, *open circles*). TSG-6 readily forms covalent complexes with HC during 120 min of premixing with IαI, whereas the HC·TSG-6 concentration without premixing at the point of addition to HA should be extremely small (21).

In both assays, a steady increase in the amount of adsorbed material was observed. Comparison of the initial binding rates in Figs. 3*A* (at 23 min) and 1*B* (at 36 min) reveals that binding from the premixed IαI/TSG-6 solutions is slowed down significantly, by 2–4-fold, compared with TSG-6 alone. This means that in a mixture with IαI, the HA binding properties of TSG-6 are impaired, which is in agreement with the (partial) displacement that we had observed in the sequential incubation assay (Fig. 1*B*).

Comparison of the two curves in Fig. 3*A* shows that the 120-min premixing of TSG-6 and IαI has an appreciable effect on the subsequent interaction with HA. First, the binding rates in the 120-min premix scenario were significantly slower than without premixing throughout the entire binding process. Second, the stability of binding was different. Following 120 min of premixing, binding was completely stable to rinsing in buffer, whereas a small but significant fraction was released without premixing. As in the sequential binding assay, sizeable fractions could be eluted by exposure to 2 and 8 m GuHCl. The remainder of the film could be completely removed by digestion with HA-ase, confirming that all remaining protein material was covalently bound to HA. If the covalently bound protein fraction consists exclusively of HCs, then the maximal occupancy after 2 h of incubating HA with the protein mixture is 35 HCs/HA chain.

To shed light on the molecular species present in the film, the fractions eluted through exposure to 2 and 8 m GuHCl or digestion by hyaluronidase were subjected to Western blot analysis with anti-TSG-6 and anti-IαI antibodies (Fig. 3*B*). All samples eluted in GuHCl showed a band with an apparent molecular mass of ∼36 kDa, identical to TSG-6 protein in a control sample taken from a premixed solution of TSG-6, HA_14_, and IαI (21). Two bands at ∼100 kDa were detected by both antibodies in the GuHCl-eluted fractions and the control, consistent with the presence of covalent complexes of TSG-6 with HC1 or HC2 (21). In addition, a minor band at ∼65 kDa was detected with RAH-1, which we suggest is a disulfide-linked TSG-6 dimer, because we know this sometimes forms during electrophoresis when TSG-6 is reduced with β-mercaptoethanol.^6^ In addition to HC·TSG-6 complexes, a rather large number of bands were stained exclusively with the anti-IαI antibody. In particular, bands for intact IαI and isolated heavy chains are clearly visible at ∼190 and 80 kDa, respectively. Notably, the same bands were detected regardless of the premixing time of IαI and TSG-6, albeit at different intensities. Apparently, the premixing time does not affect the identity, but rather the quantity, of material incorporated into the HA film.

As already observed in the sequential binding assay, no bands were detected with the anti-TSG-6 antibody in HA-ase digests, whereas the strongest band visible with anti-IαI antibody ran at ∼80 kDa. This suggests that the major, and perhaps only, covalently incorporated species are heavy chains. Our findings provide evidence that although TSG-6 is always active to transfer HC into HA films, the efficiency of HC transfer and/or incorporation of other protein species depends on the conditions of co-incubation.

**FIGURE 4.**
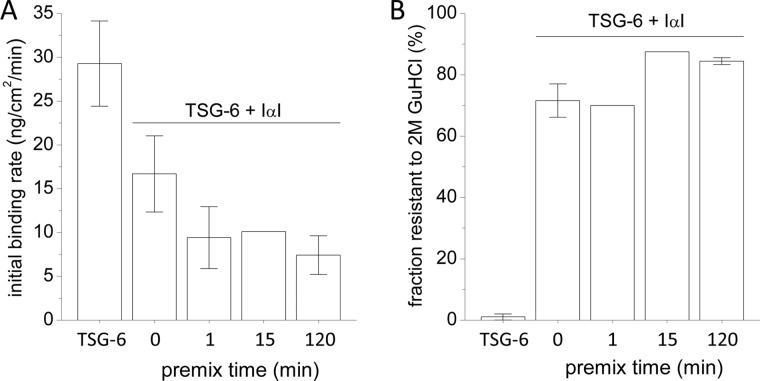
**Ternary interaction of HA, TSG-6, and IαI as a function of TSG-6/IαI premixing time.** TSG-6 at 0.3 μm was exposed to HA (837 kDa) films either alone or in a mixture with 1 μm IαI for 2 h. Proteins were premixed *ex situ* for different periods of time (0–120 min). *A*, initial rate of binding to the HA film. *B*, protein fractions that were resistant to elution in 2 m GuHCl, as percentages of total bound protein. The data plotted correspond to one measurement or to the mean of two or three independent measurements (with *error bars* indicating maximal/minimal measured values).

Intrigued by the large differences observed between the co-incubation assays with 120 min and those without premixing (Fig. 3*A*), we tested how the premixing time influences the initial binding rate of protein into the HA film (Fig. 4). With increasing premixing time, the initial binding rate of the TSG-6/IαI mixture decreased rapidly (Fig. 4*A*). Already after 1 min of premixing, the initial binding rate had reached levels comparable to those observed for 120 min. The fraction of very tightly bound material (*i.e.*, stable to 2 m GuHCl), on the other hand, was only influenced to a small extent; *i.e.*, with ∼70% bound at 0 and 1 min premixing time and ∼85% at 15 and 120 min (Fig. 4*B*). Apparently, there is a short time window for premixing, not longer than a few minutes, that influences the amount of incorporation of material into HA films.

##### IαI Binding and HC Transfer Activity Are Retained, Albeit Reduced, in the Absence of TSG-6 in the Soluble Phase

**FIGURE 5.**
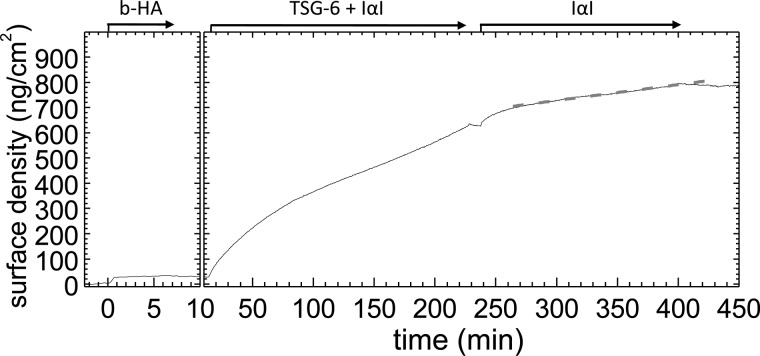
**HA-bound material is catalytically active.** An HA (1,083 kDa) film was first exposed to 0.3 μm TSG-6 and 1 μm IαI (1 min premixing) for 4 h, and residual proteins in the soluble phase were then removed by buffer exchange. All incorporated material remained stably bound in the HA film. Addition of 1 μm IαI, without TSG-6, induced significant binding. Beyond 15 min of incubation and for at least 2 h, the adsorbed mass increased linearly at a slow rate (the *dashed line* is a fit), suggesting HC transfer. *b-HA*, biotinylated HA.

To test whether HC transfer is exclusively driven by TSG-6 in the soluble phase, an HA film was first exposed to a premixed (1 min) solution of IαI and TSG-6 (Fig. 5, at 15 min) for 210 min. IαI and TSG-6 were then removed from the bulk solution by rinsing in buffer, and 1 μm IαI was subsequently added without TSG-6 (Fig. 5, at 240 min). Despite the absence of TSG-6 in the solution phase, we observed a significant increase in the areal surface density, confirming binding of IαI into the HA film. Over 2 h of incubation, the areal surface density continued to increase. Except for a faster initial phase of ∼15 min, the areal surface density increased linearly, at a rate of 0.6 ng/cm^2^/min, *i.e.*, somewhat lower than the rates observed in Figs. 1*B* and 3.

The linear trend and the low rate suggest that this phase corresponds to enzymatic transfer of HCs, implying that HA-bound TSG-6 material has a catalytic function on its own. This and the significantly lower rate in the absence of TSG-6 in solution indicate that HC transfer can occur via two different pathways, *i.e.*, from HC·TSG-6 complexes in solution that interact very weakly with HA, and via protein complexes (presumably TSG-6 and/or HC·TSG-6) that interact stably yet in a noncovalent manner with HA or with HCs that are covalently attached to HA.

##### Effect of IαI Incubation on the Thickness of the HA Film

**FIGURE 6.**
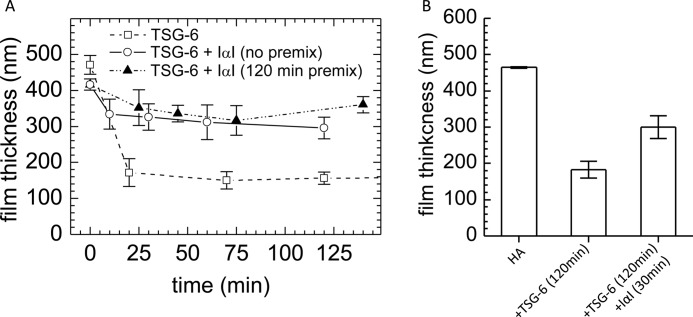
**Changes in the thickness of HA films upon incubation with TSG-6 and IαI.** Variations in the thickness of a HA (1,083 kDa) film as a function of incubation time were quantified by colloidal probe RICM. *A*, incubation with a mixture of 0.3 μm TSG-6 and 1 μm IαI (no premixing, *open circles*; 120 min premixing, *filled triangles*) resulted in a minor decrease in film thickness during the first 25 min after incubation. In contrast, incubation with TSG-6 alone, at the same concentration, induced pronounced condensation of the film within the same time (*open squares*). Film thicknesses remained virtually unaltered throughout the subsequent 2 h of incubation. *B*, addition of 1 μm IαI to the TSG-6-loaded film for 30 min led to a partial recovery of the original film thickness. The data are plotted as mean values of 10 independent measurements on the same surface (± S.E.). The film thickness did not change significantly upon prolonged incubation of IαI (*i.e.*, for a total of 120 min; data not shown).

Next, we studied how the covalent modification of HA films by HCs influences the morphology of HA. We had reported earlier that TSG-6, when presented alone, cross-links HA and induces HA film shrinkage (4). Indeed, addition of TSG-6 at 0.3 μm resulted in a strong decrease of the film thickness by more than 60% (Fig. 6). In contrast, the thickness of the HA film decreased only ∼20%, when TSG-6 was co-incubated with 1 μm IαI. Apparently, the cross-linking properties of TSG-6 are impaired in the presence of IαI. Beyond 30 min of incubation, the thickness remained virtually constant (Fig. 6*A*). The TSG-6/IαI premixing time had no significant influence on the film thickness in the co-incubation assays. However, the film only partially recovered, to 65% of its original thickness, when an already TSG-6 cross-linked HA film was exposed to IαI (Fig. 6*B*).

##### Effect of IαI on TSG-6-enhanced HA Cell Coats

**FIGURE 7.**
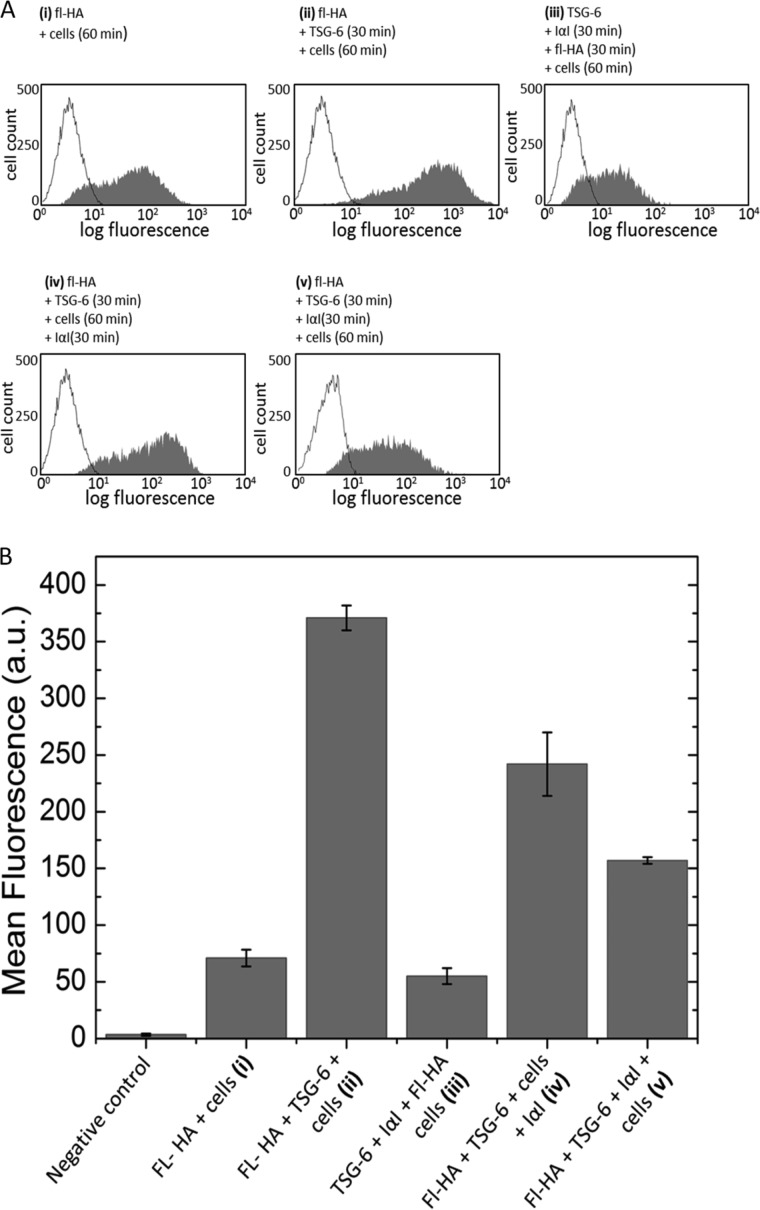
**The effect of IαI on TSG-6 enhancement of HA binding to CD44.** *A*, representative histograms showing the distribution of the fluorescence of 10,000 cells for fl-HA binding to CD44 in the absence of TSG-6 (*sample (i)*), in the presence of TSG-6 (*sample (ii)*), and in the presence of TSG-6 together with IαI (*samples (iii)* and *(v)*). TSG-6 and IαI in samples (*sample (iii)* and *(v)*) were incubated together at different sample preparation stages. In *sample (iii)*, the TSG-6 was incubated with IαI prior to the addition of fl-HA and cells. In *samples (iv)* and *(v)*, TSG-6 was incubated with HA. IαI was subsequently added after the addition of cells in *sample (iv)*, *i.e.*, after the formation of a TSG-6 enhanced film, and before the addition of cells in *sample (v)*. Final concentrations of 1 μg/ml fl-HA, 0.3 μm TSG-6, 1 μm IαI, and ∼5 × 10^5^ AKR1/CD44+ positive cells were used throughout; fl-HA, TSG-6, and IαI were incubated for 30 min, and cells were incubated for 60 min, all at 37 °C. *B*, mean fluorescence intensity for the negative control sample (*i.e.*, PBS alone) and for each sample (*samples (i)–(v)*; defined above). IαI reverses the enhancement of the CD44/HA interaction induced by TSG-6 in all assays, yet the degree of reversal depends on the incubation sequence. The data are plotted as means of three independent experiments performed in triplicate (± S.E.).

Lesley *et al.* (52) have shown that TSG-6 can act as a strong enhancer of HA binding to the surface of CD44 positive cells. In light of the drastic impact of IαI on the binding of TSG-6 to HA (Figs. 1 and 3) and on the morphology of the HA films (Fig. 6), we hypothesized that IαI would also affect the enhanced binding of HA to cells. To test this, we adapted the assay by Lesley *et al.* (52) using a CD44+ cell line that constitutively binds HA and quantified the influence of TSG-6 and IαI on the binding of fluorescently labeled HA (fl-HA) to AKR1/CD44+ cells (Fig. 7); in control experiments (data not shown), AKR1/CD44+ cells were demonstrated to exhibit surface CD44 expression and HA binding properties equivalent to those reported previously (52).

**FIGURE 8.**
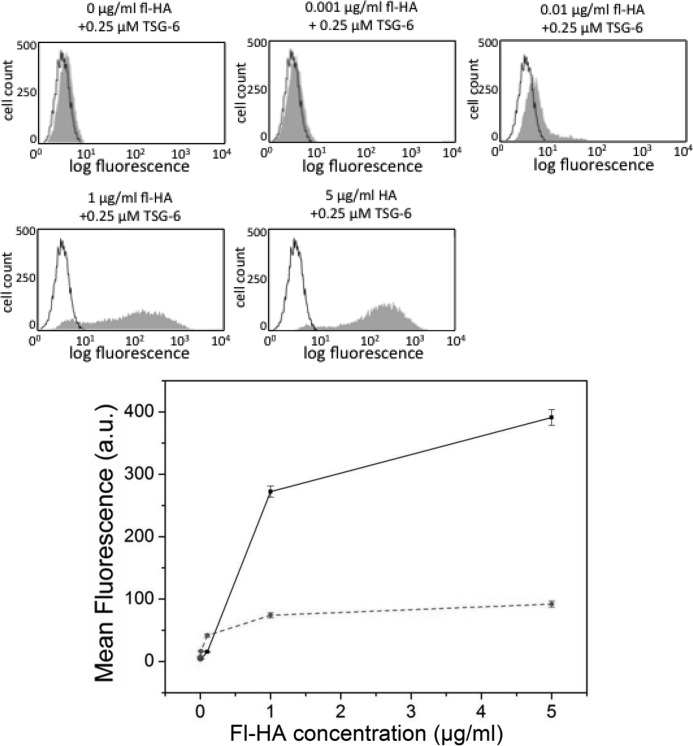
**Dose-dependent TSG-6-mediated enhancement of fl-HA binding to CD44.** AKR1/CD44+ cells were incubated with 0–5 μg/ml fl-HA in the absence or presence of 0.25 μm TSG-6, and the fluorescence intensity of ∼10,000 cells was analyzed. Representative HA binding data are shown (in *gray*), and the no fl-HA (negative control) is depicted as a *black outline*. Mean fluorescence values were calculated for fl-HA incubated in the absence (*dashed red line*) and presence (*solid black line*) of TSG-6 from three independent experiments performed in triplicate and plotted (± S.E.) in the *lower panel*.

The presence of TSG-6 alone strongly enhanced HA binding to AKR1/CD44+ cells (compare *samples (i)* and *(ii)* in Fig. 7*B*), which is in good agreement with Lesley *et al.* (52). This effect of TSG-6 was HA dose-dependent (Fig. 8). In contrast, when fl-HA was incubated with a premixed solution of TSG-6 and IαI and then added to the cells (*sample (iii)*), the mean fluorescence intensity was comparable to that observed for fl-HA alone (*sample (i)*). Hence, co-incubation with IαI is sufficient to inhibit any enhancement of the CD44/HA interaction by TSG-6. Following addition of IαI to TSG-6/HA-coated cells (*sample (iv)*), a 35% decrease was observed in the mean fluorescence, indicative of a partial reversal of the enhancement of the CD44/HA interaction induced by TSG-6. The mean fluorescence intensity of cells exposed to preformed TSG-6/fl-HA complexes that had been premixed with IαI (*sample (v)*) was intermediate between fl-HA alone and TSG-6/fl-HA, giving a clear indication that IαI is able to partially reverse the TSG-6/HA complex formation. Taken together, the effect of IαI on the TSG-6-enhanced binding of HA to CD44+ cells mirrors very closely the IαI-mediated impairment of TSG-6 binding into HA brushes.

## DISCUSSION

The ternary interactions between HA, TSG-6, and IαI were investigated in an ultrastructural context by using films of end-grafted HA and surface-sensitive analysis techniques. The HA films are well defined in that their surface density and thickness can be controlled. Time-resolved measurements by *in situ* ellipsometry and RICM provided quantitative information about the kinetics of protein incorporation/release and concomitant changes in film thickness, respectively. A combination of several purpose-designed assays, sequential addition and co-incubation, revealed several different interactions and provided novel insight into the interaction mechanisms at play. The key findings of this study are: (i) In the presence of IαI, TSG-6 is partially displaced from HA films (Fig. 1), and HA cross-linking through HA-induced TSG-6 oligomers is impaired (Fig. 6). In particular, this effect leads to impairment of TSG-6 enhanced binding of HA to CD44 positive cells (Fig. 7). (ii) Long term incubation of HA with TSG-6 and IαI leads to films that contain, in addition to covalently HA-bound HCs, several tightly but noncovalently bound molecular species. A likely route of incorporation is through the strong noncovalent interaction of TSG-6 (or HC·TSG-6) with HC1 and HC2 (Fig. 2). The timing of the encounter between TSG-6, IαI and HA has an appreciable effect on the ultimate film composition (Figs. 3 and 4). (iii) TSG-6, or TSG-6-related species, can drive the formation of HA·HC complexes both from the soluble phase and when stably bound in the HA film (Fig. 5).

### 

#### 

##### A Hierarchy of Interactions Determines Protein Function

The present study, together with previously published work from ourselves and others, provides compelling evidence that a variety of different complexes can be generated upon interaction of TSG-6, IαI (or its subunits), and HA. Among them are two covalent complexes, namely HC·HA (6) and HC·TSG-6 (21, 22), and many noncovalent complexes of varying affinity, including HA-TSG-6 (29, 32, 33), HC-TSG-6 (this study), HC-HC (7), TSG-6-TSG-6 (4), C4S-TSG-6 (25, 53–55), and TSG-6-bikunin (23, 25, 56, 57).

Here, we show that many of these complexes can be present at the same time, giving rise to supramolecular matrices of complex composition. Remarkably, the presence of IαI switches the function of TSG-6 from being an HA cross-linker to being an enzyme and in part a stably incorporated matrix component, where the sequence of encounter between the different molecular species has an appreciable effect on matrix composition.

Based on these observations, we propose that there is a hierarchy of interactions between the molecular players that ultimately determines protein functions as well as matrix assembly. This concept may not be restricted to the three molecules investigated here but can perhaps be extended to additional molecular species. For example, pentraxin 3 (PTX3) is known to be crucial for COC matrix assembly in addition to HA, TSG-6, and IαI and interacts with TSG-6 and IαI (10, 11, 13), so will likely add further complexity/regulation to this system.

Future studies that aim at mapping the hierarchy of interactions will be needed to gain a full mechanistic understanding of the regulation of matrix assembly. Based on currently available data, we hypothesize that HC·HA complexes play a central role in matrix assembly, because (i) they serve as docking sites for the attachment of other proteins and protein complexes to HA chains and (ii) they may also be involved in matrix stabilization.

##### Possible Mechanisms for the IαI-induced Displacement of TSG-6 from HA

It was demonstrated before that the interaction between TSG-6 and IαI proceeds in two steps: first, the noncovalent binding of TSG-6 to bikunin-chondroitin sulfate and second the cation-dependent transesterification in which the ester bond between HC and C4S in IαI is transferred to Ser-28 of TSG-6 located in the N-terminal region, next to the Link module, with generation of a covalent HC·TSG-6 complex (23, 25). In contrast, the recombinant Link module of TSG-6, lacking the N-terminal region, was found to bind to IαI (and to potentiate the anti-plasmin activity of the bikunin chain), but without formation of a covalent complex with HC (56, 57). Notably, the Link module of TSG-6 has binding sites for both HA and bikunin·C4S, which are likely to overlap based on data from competition experiments and their mapping by site-directed mutagenesis (53, 56). Thus, it appears possible that the competition between HA and bikunin·C4S for the Link module drives, or contributes to, the release of TSG-6 from HA through IαI (Fig. 1).

Here, we show that TSG-6 can also bind noncovalently to HC1, HC2, and HC3 (Fig. 2). These interactions occur with higher affinity (*K_D_* = 10 nm) than its binding to HA (*K*_0.5_ = 1 μm (4)) or to bikunin·C4S (*K_D_* = 180 nm (25)). It is possible, therefore, that TSG-6 may be able to bind simultaneously to bikunin·C4S and a HC within IαI, which would lead to the formation of a very stable complex (*e.g.*, with a combined affinity of up to *K_D_* = 2 fM). However, preliminary results (data not shown) indicate that IαI binds with a similar affinity to that of the HCs (∼10 nm); this indicates that TSG-6 can form a number of different complexes with IαI via individual interactions with HC1, HC2, or bikunin·CS. If any of these interactions occlude the HA-binding site on TSG-6 (or perhaps stabilize it in its closed conformation, see refs (32, 33)), this could provide a mechanism to explain why the presence of IαI so rapidly inhibits TSG-6-HA binding.

Based on the tight interaction of HCs with TSG-6, we propose a new two-stage model for HC·TSG-6 formation: the first stage would involve the metal ion-independent interaction of TSG-6 with HC1 or HC2 (and perhaps even bikunin·CS) into a very stable IαI-TSG-6 complex; this would then be followed by a metal ion-dependent transesterification step leading to HC·TSG-6.

##### The Nature and Activity of Noncovalently Matrix-bound TSG-6

The finding that the interaction between IαI and TSG-6 in the presence of HA can give rise to TSG-6 that is stably but noncovalently bound to HA and that some of this material can interact with IαI and most likely also transfer HCs to HA is novel. Colón *et al.* (58) had previously reported that HA-bound TSG-6 can mediate transfer of HCs to HA, using a sequential incubation plate binding assay with immobilized HA. In that study, however, TSG-6 was found to form nondissociable complexes with HA, *i.e.*, these complexes are likely to be different from the noncovalently HA-bound material in our study. The nondissociable TSG-6·HA complexes were formed at a nonphysiological ionic strength of 500 mm NaCl even in the absence of IαI (59), whereas we found all TSG-6 that was incorporated into HA films under physiological ionic strength to be dissociable (Fig. 4*B*) (4). It is therefore unlikely that the HC transfer pathway reported by Colón *et al.* (58) occurs in our HA films.

##### Covalent Modification of HA with HCs

In our 2-h co-incubation assays (Fig. 3), the amount of covalently incorporated material reached values corresponding to up to 35 HCs per HA chain. For comparison, three to five HCs per HA chain were found in the synovial fluid of arthritis patients (7). The size of HA in synovial fluid is approximately double the size in our HA films, *i.e.*, the HC coverage in our films is 14–24-fold higher than in this *in vivo* situation.

Given that both HA and IαI are present in synovial fluid of arthritis patients and that the HA concentration in synovial fluid (between 1 and 4 mg/ml (60)) is comparable to our HA films (1 mg/ml (44)), a plausible explanation for the difference could be that the extent of covalent modification correlates with TSG-6 concentration. In this case, a concentration of 16 nm TSG-6 would be enough to transfer four HCs per HA chain in 2 h. This number correlates rather well with the concentrations of TSG-6 (up to 3 nm) recently reported for synovial fluids of patients with inflammatory arthritis (61).

##### IαI- and TSG-6-mediated Cross-linking

In earlier work, we demonstrated that TSG-6 alone can cross-link and induce condensation of HA into dense matrices (4). Here, we show that TSG-6-mediated condensation does not occur in the presence of IαI and can even be reversed upon addition of IαI to HA/TSG-6 complexes.

*In vivo*, the ternary interaction of IαI, TSG-6, and HA is regulated by the temporal expression and localization of reactants. The synthesis of HA and TSG-6 is often coordinated and occurs in tissue extracellular matrix during inflammation. In contrast, IαI is constitutively present in the blood. It can only diffuse into tissue extracellular space when the permeability barriers that separate different tissues from blood become leaky, for instance during vasodilation or ovulation. This raises the possibility that a dense TSG-6/HA matrix (with enhanced CD44 binding properties) may form in the absence of IαI (early in inflammation) and that this would be reversed as soon as IαI penetrates into the tissue, providing a mechanism to regulate HA-mediated cell signaling and perhaps promote tissue swelling. In osteoarthritic cartilage, for example, TSG-6 is present (62), in the absence of intact IαI (63), in the deep zones of the tissue, representing a site where cross-linked TSG-6/HA complexes could form. Closer to the articular surface, HA/TSG-6 complexes would be exposed to IαI present in the fibrillated cartilage, which might be envisaged to modulate the organization of the HA matrix.

Based on the results presented here, it appears unlikely that TSG-6 oligomers have a direct structural role as cross-linkers in the expanded COC matrix (*i.e.*, given the presence of IαI). Thus, some other type of cross-link must be utilized to form a cohesive matrix. Cross-linking via HCs has been proposed as another possible HA cross-linking mechanism (either via HC-HC interactions between HC·HA (7) and/or via interactions between HC·HA and PTX3 (11, 13), with PTX3 being essential in the case of COC matrix stabilization (10, 11)). In general, one would expect the introduction of (sufficiently stable) cross-linkers to force neighboring HA chains in an expanded meshwork closer together, leading to condensation. Our measurements of the film thickness (Fig. 6) indicate only a minor condensation in the presence of IαI. Moreover, the thickness decrease occurred within less than 30 min (Fig. 6), whereas HC incorporation continued over many hours (Figs. 1, 3, and 5). The small decrease in thickness and the limited correlation between thickness decrease and HC incorporation suggest that cross-linking via HC-HC interactions between HC·HA, if present at all, is rather weak. We can, however, not exclude the possibility that the presence of noncovalently bound material somehow prevents this type of cross-linking from occurring. Further studies are clearly required to fully elucidate the mechanisms behind COC matrix stabilization, including the role of PTX3.

##### Role of HC·HA in Leukocyte Adhesion

Here we have found that TSG-6-mediated transfer of HCs onto HA counteracts the enhancement of binding seen with TSG-6/HA complexes and does not promote the binding of HA to CD44+ cells. This is in stark contrast to a previous finding that HC·HA binds better to CD44+ leukocyte cell lines when compared with unmodified HA (16). One major difference between the two studies is that whereas we formed HC·HA *in vitro* (with purified human IαI (19), recombinant TSG-6 (42), and medical grade HA), the material used by Zhuo *et al.* (16) was isolated from the synovial fluids of patients with rheumatoid arthritis. Thus, although the HC·HA used in the present study will only contain HC1 and HC2 (and possibly noncovalently bound TSG-6/HC·TSG-6 species), the native material (16) is likely to contain HC3 (6) along with HA-binding proteins, such as PTX3, which is known to be present in rheumatoid synovial fluid (64); interestingly, the purified HC·HA was found to be free of any associated TSG-6 (16). It seems likely therefore that compositional differences may explain the distinct adhesive properties of these two different HC·HA preparations. This raises the exciting possibility that the exact composition of the inflammatory milieu can regulate HA-receptor interactions and hence determine the extent of cell adhesion and HA-mediated signaling.

Taken together, this study strikingly illustrates how hyaluronan, although structurally a very simple molecule, can promote many different processes and functions, depending on the microenvironment or availability of particular binding partners. Thus, our results provide an important new insight as to how the broad range of HA biology arises from the complexity and diversity of HA-protein interactions.

## Footnotes

5 D. C. Briggs, C. M. Milner, and A. J. Day, unpublished data.

6 A. J. Day, unpublished data.

## Footnotes

4 The abbreviations used are:

HA: hyaluronan
fl-HA: fluorescein labeled HA
HC: heavy chain
rHC: recombinant human heavy chain
IαI: inter-α-inhibitor
COC: cumulus-oocyte complex
C4S: chondroitin 4-sulfate
SAv: streptavidin
GuHCl: guanidine hydrochloride
RICM: reflection interference contrast microscopy
PTX3: pentraxin 3
HA-ase: hyaluronidase.
